# Towards Lipid from Microalgae: Products, Biosynthesis, and Genetic Engineering

**DOI:** 10.3390/life14040447

**Published:** 2024-03-28

**Authors:** Yi Xin, Shan Wu, Congcong Miao, Tao Xu, Yandu Lu

**Affiliations:** 1 State Key Laboratory of Marine Resource Utilization in South China Sea, School of Marine Life and Aquaculture, Hainan University, Haikou 570228, China; wushan@hainanu.edu.cn (S.W.); ccmiao@hainanu.edu.cn (C.M.); xtao@hainanu.edu.cn (T.X.); 2 Haikou Technology Innovation Center for Research and Utilization of Algal Bioresources, Hainan University, Haikou 570228, China; 3 Hainan Provincial Key Laboratory of Tropical Hydrobiotechnology, Hainan University, Haikou 570228, China

**Keywords:** alga, microalgae, lipids, fatty acids, triacylglycerol

## Abstract

Microalgae can convert carbon dioxide into organic matter through photosynthesis. Thus, they are considered as an environment-friendly and efficient cell chassis for biologically active metabolites. Microalgal lipids are a class of organic compounds that can be used as raw materials for food, feed, cosmetics, healthcare products, bioenergy, etc., with tremendous potential for commercialization. In this review, we summarized the commercial lipid products from eukaryotic microalgae, and updated the mechanisms of lipid synthesis in microalgae. Moreover, we reviewed the enhancement of lipids, triglycerides, polyunsaturated fatty acids, pigments, and terpenes in microalgae via environmental induction and/or metabolic engineering in the past five years. Collectively, we provided a comprehensive overview of the products, biosynthesis, induced strategies and genetic engineering in microalgal lipids. Meanwhile, the outlook has been presented for the development of microalgal lipids industries, emphasizing the significance of the accurate analysis of lipid bioactivity, as well as the high-throughput screening of microalgae with specific lipids.

## 1. Introduction

Microalgae are autotrophic and unicellular organisms that grow in aquatic environments. They are at the bottom of the food chain and involved in carbon and biochemical cycles. Microalgae are one of the main producers of lipids, which are up to ten times higher than their counterparts in terrestrial plants [1,2]. Many microalgal species, such as *Dunaliella salina*, *Phaeodactylum tricornutum*, and *Nannochloropsis oceanica*, can produce up to 70% of total lipids in biomass [3]. In microalgae, neutral lipids are an important source of energy, which can be applied for the production of biofuel and biodiesel. On the other hand, polar lipids usually exhibit biological activity. Moreover, microalgal lipids can reduce inflammation, support heart function and brain health, and even prevent cancer, obesity, and Alzheimer’s disease [4].

Microalgal lipids are a class of organic compounds, which can be used as raw materials for food, feed, cosmetics, healthcare products, bioenergy, etc., with tremendous potential for commercialization [5]. In this review, we summarized the commercial lipid products from eukaryotic microalgae, and analyzed the synthesis mechanisms of lipids in microalgae. Moreover, we reviewed the enhancement and/or modification of lipids, triacylglycerols (TAGs), polyunsaturated fatty acids (PUFAs), pigments, and terpenes in microalgae via environmental induction and/or metabolic engineering in the past five years. Collectively, we provided a comprehensive overview of the products, biosynthesis, and genetic engineering in microalgae. Meanwhile, the outlook has been shown for the development of microalgal lipids industries, emphasizing the significance of the accurate analysis of lipid bioactivity, as well as the high-throughput screening of microalgae with specific lipids.

## 2. Application of Lipids from Microalgae

Microalgae-derived lipids, as their multi-functions in human health, can be applied by a series of compound species including PUFAs, TAGs, carotenoids, etc. (Table 1). Microalgae are impressive as the only photosynthetic organisms that produce omega-3 PUFAs, including eicosapentaenoic acid (EPA) and docosahexaenoic acid (DHA) [6]. PUFAs are essential components of a healthy diet. Epidemiological and clinical studies have shown that an EPA-rich diet is beneficial to minimize risks of cardiovascular diseases [7]. Arachidonic acid (ARA) and DHA are important to avoid impairments in infant cognitive deficiency and brain development [8]. EPA and DHA were considered to prevent chronic inflammatory diseases and lower the risks of obesity [9]. Humans, like other mammals, are unable or poorly able to synthesize some essential PUFAs, such as linoleic acid (LA) and *α*-linolenic acid (ALA), which are precursors of ARA and DHA, respectively [8]. Compared to fish oil, microalgal PUFAs contain lower levels of dioxins, methyl mercury, and polychlorinated biphenyls [10,11]. In addition, fish oil usually results in allergy [3]. Thus, microalgal PUFAs play a key role in the economical production of pharmaceuticals, cosmetics, nutrients, and food [10,12].

In microalgae, TAG composes up to 60% of dry weight. TAG is a main form of microalgal energy storage [13]. The TAG molecule harbors three fatty acid (FA) moieties that are anchored to a glycerol scaffold. The diversity and sn-location of these TAG-associated FAs are key properties to determine the application area, economic value, and market potential of microalgal oil products. On one hand, medium-chain triglycerides (MCTs) contain medium-chain FA (MCFA) esterified to the glycerol backbone. These MCFAs have a shorter chain length and are quickly metabolized in the body, serving as an immediate energy source. They are known to have good physiological as well as functional characteristics which help in treating various health disorders [14]. On the other hand, 1,3-dioleolyl-2-palmitate (OPO) is an important component of human milk fat. Its unique FA composition and distribution play an important role in proper infant growth and development [15]. Interestingly, MCT and OPO have been identified in microalgal species, such as *N. oceanica* [16] and *Chlamydomonas reinhardtii* [17].

Carotenoids are liposoluble pigments, exhibiting antioxidant features. The content of microalgal carotenoids is much higher than the counterpart of land plants. Under high temperature or light intensity, microalgae can synthetize a large number of carotenoids, preventing the damage of free radicals [18]. Thus, carotenoid-enriched microalgae are considered as valuable feedstocks in the healthcare and pharmaceutical industries [19]. Commercial carotenoids comprise fucoxanthin, astaxanthin, lutein, β-carotene, canthaxanthin, zeaxanthin, neoxanthin, and lycopene. Besides carotenoids, microalgal phytosterols are another class of lipid compounds with intriguing bioactive properties. Phytosterols have been found to decrease total cholesterol by hindering intestinal absorption [20]. In *Chlorococcum* sp., PUFA-containing phosphatidylcholine has been characterized to be agonistic and antagonistic to the platelet-activating factor pathway in human platelet aggregation [21].

**Table 1:** Performance of microalgae species for bioactive lipid production and its functions. * N/A, not available; ** $, U.S. dollar; *** DW, dry weight.

Genus	Lipid Yield (% DW ***)	Bioactive Lipid	Market Price (** $/g)	Application	References
*Chlorococcum* sp.	20–24	Phosphatidylcholine	50–8000	Anti-inflammatory, anti-thrombotic activities	[21]
*Nannochloropsis* spp.	37–60	Eicosapentaenoic acid	40–23,000	Reduce heart attack and cardiovascular death	[22]
*Crypthecodinium cohnii*, *Schizochytrium* spp.	14–33	Docosahexaenoic acid	2–4000	Improved vision, brain, and memory development	[23]
*Chlamynodomonas reinhardtii*	25–51	1,3-dioleolyl-2-palmitate	4–16,000	Proper infant growth and development	[15,17]
*Nannochloropsis oceanica*	23–68	Medium-chain triglyceride	3–16,000	Anti-atherosclerosis, anti-obesity	[14,16]
*Phaeodactylum tricornutum*	10–32	Fucoxanthin	1000–43,000	Ophthalmic, cerebrovascular and hepatic health	[24,25]
*Euglena gracilis*	9–17	Lycopene	2–5300	Antioxidant, cerebrovascular health	[26]
*Coelastrella terrestris*	11–23	Canthaxanthin	1–20,000	Antioxidant, visual health	[27]
*Heterosigma akashiwo*	N/A *	Zeaxanthin	1–110,000	Anti-Inflammatory, anticancer	[28]
*Chlamydomonadales* sp.	15–23	Neoxanthin	5000–120,000	Antioxidant, cardiovascular health	[29]
*Haematococcus pluvialis, Chlorella zofingiensis*	30–50	Astaxanthin	3–4500	Anti-oxidation, anti-inflammation	[30,31]
Rhodophyte, Chlorophyte, Bacillariophyte, etc.	12–33	Oxylipins	1–50,000	Anti-inflammatory, tissue regeneration	[32]
*Chlorella protothecoides*	10–30	Lutein	1–27,000	Immune stimulant, anti-inflammatory, antioxidant	[33]
*Dunaliella salina*	12–44	β-carotene	1–12,000	Antioxidant, anti-allergic, anti-inflammatory	[33,34]

## 3. Lipid Biosynthesis in Microalgae

**Figure 1:**
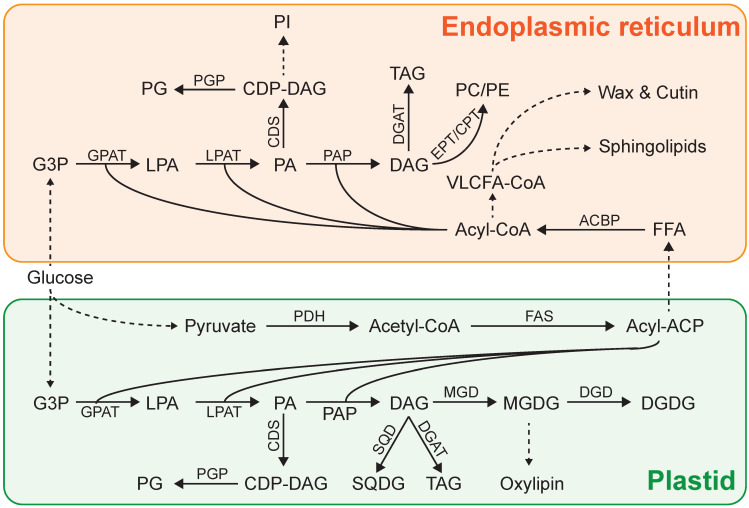
Mechanistic model of lipid assembly lines in eukaryotic microalgae. Not all intermediates or reactions are displayed. Arrows indicate catalytic steps in the pathway. ACBP, acyl-CoA-binding protein; ACP, acyl carrier protein; CDP-DAG, cytidine diphosphate-diacylglycerol; CDS, cytidine diphosphate-diacylglycerol synthase; CPT, cholinephosphotransferase; DAG, diacylglycerol; DGAT, diacylglycerol acyltransferase; DGD, digalactosyl dehydrogenase; DGDG, digalactosyl diacylglycerol; EPT, ethanolamine phosphotransferase; FAS, fatty acid synthase; FFA, free fatty acid; G3P, glycerol-3-phosphate; GPAT, glycerol-3-phosphate acyltransferase; LPA, lysophosphatidic acid; LPAT, lysophosphatidic acid acyltransferase; MGD, monogalactosyl dehydrogenase; MGDG, monogalactosyl diacylglycerol; PA, phosphatidic acid; PAP, phosphatidic acid phosphatase; PC, phosphatidyl choline; PDH, pyruvate dehydrogenase; PE, phosphatidyl ethanolamine; PGP, phosphatidylglycerol phosphate; PI, phosphatidylinositol; SQD, sulfoquinovosyldiacylglycerol dehydrogenase; SQDG, sulfoquinovosyldiacylglycerol; TAG, triacylglycerol; VLCFA, very long-chain fatty acids.

Lipids are produced by two plastid pathways plus one nuclear pathway (Figure 1). The plastid acetate pathway is in charge of the de novo synthesis of FAs, as well as the derivatives, such as alkanes and fatty alcohols [35]. Terpenoids, including carotenoids and sterols, are also synthesized in the chloroplast by the methylerythritol phosphate deoxy-xylulose phosphate (MEP-DOXP) pathway [36]. Acetate is used for hydrocarbon elongation and successive condensation. Meanwhile, acetate is also used to generate dimethylallyl pyrophosphate, isopentenyl pyrophosphate, and 5-carbon units, which are also generated in the cytosol by the 3-hydroxy-3-methyl-glutaryl-coenzyme-A reductase pathway in nuclear pathways [37].

In the de novo FA synthesis of microalgae, the first committed step is acetyl-CoA carboxylation, producing malonyl-CoA. The two-step reaction is catalyzed by acetyl-CoA carboxylase (ACCase). The ACCase-generated malonyl-CoA is first transformed into a malonyl-acyl carrier protein (ACP) by malonyl-CoA:ACP malonyltransferase. Under the catalysis of ketoacyl-ACP synthase, malonyl-ACP is combined with an acetyl-CoA molecule to produce a 3-ketoacyl-ACP, which is subsequently reduced, dehydrated, and reduced again to form a 6-carbon-ACP, by ketoacyl-ACP reductase, hydroxyacyl-ACP dehydrase, and enoyl-ACP reductase, nominated as the multi-subunit bacterial type II FA synthase (FAS) complex [38]. The FAS reaction repeats for seven cycles until forming a C16-ACP, in most microalgae. The C16-ACP then enters three subsequent pathways: (i) acyltransferases-mediated acylation to glycerol for chloroplast lipids, (ii) KASII-mediated elongation to C18-ACP, or (iii) acyl-ACP thioesterase (FAT)-mediated conversion to a C16 free FA. C18-ACP can be desaturated by stearoyl-ACP desaturase to form unsaturated C18-ACPs, which are substrates of FAT. The metabolic products are then exported out of the plastid.

In microalgae, very long-chain PUFA (VLC-PUFA) can be synthetized via either aerobic or anaerobic pathway, depending on the presentation/absentation of molecular oxygen [39]. In the aerobic or oxygenic pathway, two hydrogens are removed from an acyl chain to introduce a double bond by desaturases (DESs) [40]. Most DESs exhibit high regioselectivity. For example, Δ12 and Δ15 DESs introduce double bonds toward the methyl end, while Δ5 and Δ4 DESs introduce double bonds toward the carboxyl end, respectively. On the other hand, FA elongation is promoted by an FA elongase (FAE) complex (Δ5 FAE, Δ6 FAE, Δ9 FAE, etc.) including a few discrete enzymes (e.g., ketoacyl-CoA synthase, ketoacyl-CoA reductase, enoyl-CoA reductase) [41]. The aerobic pathway can be further divided into two sub-pathways by the ω3 and ω6 families of VLC-PUFAs. In the ω6 sub-pathway, linoleic acid (LA) is metabolized by Δ6 DES, Δ6 FAE, and Δ5 DES sequentially to form ARA, and then docosapentaenoic acid (DPA), by Δ5 FAE and Δ4 DES. In the ω-3 sub-pathway, α-linolenic acid (ALA) is metabolized by Δ6 DES, Δ6 FAE, and Δ5 DES to form EPA, and then DHA by Δ5 FAE and Δ4 DES [42,43,44,45,46,47,48,49]. Meanwhile, a Δ6 FAE was found to take part in EPA biosynthesis via the ω6 pathway in *Nannochloropsis oceanica* [50]. In addition, the DHA biosynthesis goes through a retro-conversion process due to the lack of the Δ4 desaturation step, which follows two elongations of EPA to form tetracosapentaenoic acid (TPA), and a Δ6 desaturation to produce tetracosahexaenoic acid (THA) [51,52,53]. On the other hand, the anaerobic pathway initiates from a precursor acetyl thioester that is mediated by polyketide synthase (PKS)-like mega-enzyme [54], including multiple subunits, such as ketoacyl reductase (KR), ketoacyl synthase (KS), enoyl reductase, dehydratase (DH), malonyl-CoA:ACP transacylase (MAT), and ACP. These subunits coordinately synthesize VLC-PUFAs through four reactions: KS-mediated condensation, KR-mediated keto-reduction, DH-mediated dehydration, and enoyl reductase-catalyzed enoyl-reduction. Unlike long-chain FAs, VLC-PUFAs are synthetized by specific DH activity by introducing cis-double bonds. 

The synthetic pathway of glycerolipids is known as the Kennedy pathway. The de novo synthesized FAs are esterified to the backbone of glycerol-3-phosphate (G3P) by the G3P acyltransferase (GPAT) and lysophosphatidic acid acyltransferase (LPAAT) to form phosphatidic acid (PA). Meanwhile, GPAT may be involved in galactolipid biosynthesis [17]. PA enters anionic phosphoglycerides assembly, including phosphatidylinositol and phosphatidylglycerol. On the other hand, PA goes through dephosphorylation by phosphatidic acid phosphatase (PAP) to produce diacylglycerol (DAG) for further synthesis of glycosyl glycerides, including digalactosyldiacylglycerol (DGDG), sulfoquinovosyldiacylglycerol (SQDG), and monogalactosyldiacylglycerol (MGDG), or for the synthesis of zwitterionic phosphoglycerides, including phosphatidylethanolamine, phosphatidylserine, phosphatidylcholine, and betaine ether lipids in the endoplasmic reticulum (ER). DAG can be also used to form TAG by diacylglycerol acyltransferase (DGAT) [55]. Meanwhile, DAG can be transformed to TAG via the acyl-CoA-independent pathway mediated by the phospholipid:diacylglycerol acyltransferase (PDAT) [35]. In *Chlamydomonas*, MGDG is found to convert to TAG by head group removal with subsequent acylation, under N-deprivation [56].

## 4. Lipid Induction Strategies in Microalgae

Within the last 10 years, many strategies, such as high light intensity (e.g., 400 μmol photon·m^−2^·s^−1^ in *Monoraphidium dybowskii* Y2 [57]), increased CO_2_ concentration, high temperature, and nutrient limitation, have been developed to induce lipid production in microalgae (Table 2, [58]). For example, nutrient limitation, especially nitrogen starvation, is a promising strategy to control cell cycle and lipid-related pathways in microalgae [59]. However, in microalgae, the biomass or photosynthesis is usually depressed by a single strategy, due to the high concentrations of reactive oxygen species (ROS), which are mediated following cell death [60]. Thus, integrated strategies are considered to be more rational and efficient for the accumulation of lipids plus biomass in microalgae [58]. Wavelength is known to enhance the production of lipids and TAG in microalgae. In *Acutodesmus obliquus*, spectra that included wavelengths between 470 nm and 520 nm led to a significantly higher percentage of PUFAs [61]. In *Haematococcus pluvialis*, the white–red regime with C5 organic carbon showed a good potential for enhancing microalgal biomass and lipid synthesis, especially for saturated FAs. Meanwhile, the astaxanthin biosynthesis has been significantly enhanced and the highest content of 3.3% was achieved with gluconate at the white–blue regime [62]. Moreover, microwave power at 100 W, a duty cycle at 40%, and a 2 min treatment time led to a substantial improvement in the biomass and lipid content in *Scenedesmus* sp. [63]. 

Plant regulators were usually used for lipid induction. Treatment with salicylic acid induced significantly higher lipid and EPA production in *Nannochloropsis oceanica* [64], while a combination of indole acetic acid and kinetin achieved a 2.3- and 2.5-fold increase in biomass and lipid yield for *Graesiella emersonii* [65]. On the other hand, algae-associated bacteria can significantly enhance lipid production. Probiotic bacteria have been found to improve culture density, biomass, and lipid content in *Phaeodactylum tricornutum* and *Nannochloropsis oceanica* [66,67]. In addition, lipid productivity can be induced by strigolactone, phenolic compounds, and magnesium aminoclay nanoparticles in *Monoraphidium* sp., *Euglena gracilis*, and *Chlorella* sp., respectively [68,69,70]. Stress induction can also enhance lipid production, but biomass is simultaneously depressed in microalgae. Therefore, the above-mentioned factors are usually combined with stress induction to achieve dual enhancement of lipid and biomass [71,72,73,74,75,76].

In microalgae, lipid synthesis is found to be influenced by many other factors, such as ROS, nutrient supply, light intensity, temperature, CO_2_ concentration, etc. [77]. Among all influencing factors, genetic factors (i.e., key genes) play an essential role in microalgal lipid synthesis, especially in a changing environment. In the past few years, omics studies have revealed potential targets in various microalgae under different growing conditions. These works have illustrated a panoramic profile of gene expression from a series of metabolic pathways, such as RNA processing, ribosome biosynthesis, photosynthesis, protein metabolism, energy generation, TCA (tricarboxylic acid) cycle, carbon fixation, nitrogen assimilation, pentose phosphate metabolism and carbohydrate metabolism along with the enhancement in lipid accumulation under changing environments [78]. For example, a substantial increase in the transcripts of *ACP*, *DGAT*, *ACCase*, and *FAT* has been reported over an array of analyses during nitrogen starvation [79]. On the other hand, an apparent decrease in genes involved in the TCA cycle, such as malate dehydrogenase (MDH), pyruvate dehydrogenase (PDH), phosphoenol pyruvate carboxylase (PEPC), transketolase, succinyl CoA lyase, aconitase, glyceraldehyde phosphate dehydrogenase, isocitrate dehydrogenase, oxoglutarate dehydrogenase, fructose 1-6-bisphosphatase, and succinate dehydrogenase, has been widely reported under high light or high temperature [80,81].

**Table 2:** Lipid improvement by environmental factors in microalgae. * N/A, not available.

Genus	Affecting Factor	Effect to Lipid Production	Effect to Biomass	Reference
*Acutodesmus obliquus*	Blue–green light	Higher percentage of PUFAs	N/A *	[61]
*Haematococcus pluvialis*	Gluconate plus white–blue LED	Increased astaxanthin content to 3.3%	Increase to 4.5 g/L	[62]
*Scenedesmus* sp.	Microwave	Increased lipid content by 1.4 g/L	1.5-fold increase	[63]
*Nannochloropsis oceanica*	Salicylic acid	Increased lipid and EPA contents	N/A	[64]
*Graesiella emersonii*	Indole acetic acid plus kinetin	Increased lipid yield by 2.5-fold	2.3-fold increase	[65]
*Phaeodactylum tricornutum*	Marinobacter	Increased lipid content by 30 mg/L	Increase to 0.2 g/L	[66]
*Nannochloropsis oceanica*	Probiotic bacteria	Increased EPA content by 2.3-fold	1.6-fold increase	[67]
*Monoraphidium* sp.	Strigolactone	Increased lipid productivity by 55%	Increased	[68]
*Euglena gracilis*	Phenolic compounds	Increased carotenoids and lipids	2.3-fold increase	[69]
*Chlorella* sp.	Magnesium aminoclay nanoparticles	Increased lipid content by 18%	N/A	[70]
*Chlamydomonas reinhardtii*	Salt stress with NaCl and KCl	Increased saturated fatty acids	N/A	[71]
*Neochloris oleoabundans*	High light plus CaCO_3_ crystal	Increased lipid productivity by 32%	Increase to 3.1 g/L	[72]
*Scenedesmus* sp.	Oxidative stress plus nanoparticles	Increased lipid content to 40%	Increase to 3.2 g/L	[73]
*Chlorella pyrenoidosa*	Salt stress plus abscisic acid	Increased lipid productivity by 3.7-fold	1.5-fold increase	[74]
*Monoraphidium* sp.	Cu^2+^ induction plus γ-aminobutyric acid	Increased lipid content to 58%	Increase to 1.3 g/L	[75,76]
*Chlamydomonas* sp.	5% CO_2_ concentration	Increased lipid content (65%) and productivity (169 mg/L/day)		[82]
*Chlorella vulgaris*	30% CO_2_	Increased lipid content (46%) and productivity (86 mg/L/day)		[58]
*Chlorella vulgaris*	Nanoscale MgSO_4_	Increased lipid productivity by 185%		[83]
*Nannochloropsis maritima*	Fe_3_O_4_ nanoparticles	More total lipid amount	Increase to 1 g/L	[84]
*Nannochloropsis* sp.	High-light (700 μmol photons/m^2^/s)	Increased lipid content to 47%	N/A	[85]
*Scenedesmus* sp.	High-light (400 μmol photons/m^2^/s)	Increased lipid content by 11-folds	N/A	[86]
*Heterochlorella luteoviridis*	High temperature (27 °C)	Increased SFA content to 53%	N/A	[87]
*Microcystis aeruginosa*	High nitrogen (ten times higher)	Increased lipid content (34%) and productivity (47 mg/L/day)		[88]
*Chlamydomonas reinhardtii*	Limited mixotrophic conditions	66% increase in lipid production (0.08 g/L)		[58]
*Chlorella vulgaris*	MnCl_2_ (10 μM)	Increased lipid content by 16%	N/A	[89]

## 5. Genetic Engineering of Microalgae for Enhanced Lipid Production

Although microalgal wild-type s can accumulate lipids due to environmental factors, biomass productivities are usually hindered. Genetic engineering is a promising strategy to produce strains with robust lipids without growth impairment. Advances in genetic engineering and synthetic biology can facilitate current efforts to achieve an economically feasible process (Table 3). In microalgae, genetic tools such as overexpression, gene stacking, RNA interference (RNAi), homologous recombination, and clustered regularly interspaced short palindromic repeat (CRISPR) have been applied for enhanced lipid production [90]. The green model microalga *Chlamydomonas reinhardtii* emerged as a sustainable production chassis for the efficient biosynthesis of recombinant proteins and high-value metabolites [91]. To introduce carbon flux to lipid synthesis, the *PEPC1* gene was knocked down while chaperone GroELS was overexpressed in *C. reinhardtii*, resulting in the highest biomass of 2.56 g/L and also boosting the lipids and lutein with 893 and 23.5 mg/L, respectively [92]. S-adenosylmethionine (SAM) is a substance that plays an important role in various intracellular biochemical reactions, such as cell proliferation and stress response. Compared to wild-type *C. reinhardtii*, recombinant cells overexpressing *SAMS* grew 1.56-fold faster and produced 1.51-fold more lipids in a nitrogen-depleted medium. Furthermore, under saline-stress conditions, the survival rate and lipid accumulation were 1.56 and 2.04 times higher in the SAMS-overexpressing strain, respectively [93]. To channel carbon into FA synthesis, ACCase was overexpressed in *C. reinhardtii.* Under the optimized conditions, the content of lipids by overexpressing the ACCase gene in the mutant CW15-85 (0.46 g/L) was 1.16-fold greater than control [94].

FA exporters (FAXs) were found to be involved in TAG production by functioning in chloroplast and ER membranes. Overexpression of *CrFAX1* doubled the content of TAG in *C. reinhardtii* cells [95]. Co-expression of two *CrFAX*s increased the accumulation of the total lipid content in algae cells, and the FA compositions were changed under normal TAP or nitrogen deprivation conditions [96]. Moreover, co-overexpression of *CrFAX1*, *CrFAX2*, and ER-localized FA transporter (*ABCA2*) results in up to twofold more TAG than the parental strain, and the total amounts of major PUFAs in TAG increased by 4.7-fold [97,98,99]. 

To identify the regulation of *CrFAX*s, transcription factors (TFs) CrDOF and MYB1 were characterized, respectively. Overexpression of *CrDOF* in *C. reinhardtii* significantly increased the intracellular lipid content [100]. Meanwhile, *CrDOF* overexpression plus *LACS2*-*CIS* knockdown increased the intracellular lipids and FA content by 142% and 52%, whereas the starch and protein contents decreased by 45% and 24% [101]. On the other hand, *MYB1* overexpression accumulated 1.9- to 3.2-fold more TAGs, and total FAs also significantly increased. Moreover, starch and protein content and biomass production also significantly increased [102]. Knockout of *MYB1* revealed that genes involved in lipid metabolism are depressed, especially under nitrogen deficiency. Among these genes were several involved in the transport of FAs, including acyl-ACP thioesterase (FAT1), CrFAXs, and long-chain acyl-CoA synthetase1 (LACS1) [103]. Additionally, overexpression of nucleus-located *CpZF_CCCH1* downregulated genes associated with TAG assembly and lipid turnover from 2.0- to 2.9-fold, likely by binding to the GCN4 motif and promoter of *GPAT* [104]. On the contrary, CrPrp19 protein was necessary for negatively regulating lipid enrichment and cell size. Total FAs were significantly increased in CrPrp19 RNAi transformants [105].

TAG synthesis plays a key role in the lipid metabolism of *C. reinhardtii*. Overexpression of *CrGPATer* significantly enhanced galactolipids, TAG (especially OPO), and biomass of *C. reinhardtii* [17]. One *Haematococcus pluvialis LPAAT* was introduced into *C. reinhardtii*, leading to retarded cellular growth, enlarged cell size, and enhanced TAG accumulation [106]. In addition, heterogeneous expression of three *Auxenochlorella protothecoides DGAT*s increased the C18:1 content in *C. reinhardtii* CC-523 [107]. These studies provide a framework for dissecting uncharacterized DGATs, and could pave the way for decrypting the structure–function relationship of this large group of enzymes that are critical to lipid biosynthesis.

*Nannochloropsis* is a genus of fast-growing microalgae that is regularly used for biotechnology applications. *Nannochloropsis* species have high TAG content, and their polar lipids are rich in the omega-3 long-chain PUFAs, especially EPA. There is a growing interest in the *Nannochloropsis* species as a model for the study of microalga lipid metabolism and as a chassis for synthetic biology. Recently, techniques for gene stacking and targeted gene disruption and repression in the *Nannochloropsis* genus have been developed [108]. 

A systematic modification was conducted over carbon flux in *Nannochloropsis* lipid metabolism. As for the photosynthesis level, overexpression of *C. reinhardtii CAO* improved lipid productivity in *N. salina* [109]. In the carbon partition level, overexpression of *Arabidopsis thaliana DXS* results in increased lipid production by ~68.6% under nitrogen depletion and ~110.6% under high light in *N. oceanica* [110]. As for the FA synthesis, medium-chain FAs are boosted by introducing a *Cuphea palustris* acyl-ACP TE (CpTE) in *N. oceanica* [111]. Moreover, Δ12-fatty acid desaturase (FAD12) was knocked in to significantly enhance the production of linoleic acid and EPA in *N. salina* [112]. Furthermore, overexpression of *NoΔ6-FAE* reveals the involvement of NoΔ6-FAE in EPA biosynthesis via the ω6 pathway in *N. oceanica* and highlights the potential of manipulating NoΔ6-FAE for improved lipid production [50]. 

As for TAG synthesis, overexpression of glycerol-3-phosphate acyltransferase (GPAT) in *N. oceanica* had up to 51% and 24% increased TAG and PUFA contents, respectively [113]. Genetic stacking of *NoDGAT2D* with MCFA- or DAG-supplying enzymes or regulators that include *mCpTE*, *CnLPAAT*, and *AtWR1* elevates the MCT share in total TAG by 66-fold and MCT productivity by 65-fold, at the peak phase of oil production [16]. On the other hand, by overexpressing *PDAT* in *N. gaditana*, the TAG content was increased in conditions naturally stimulating strong lipid accumulation such as high light and nitrogen starvation [114]. Meanwhile, GC-MS quantification revealed that *NoPDAT* overexpression enhanced TAG by 28–33% in *N. oceanica* [115].

As for global regulation, the overexpression of a TF NobZIP1 results in a remarkable elevation of lipid accumulation and lipid secretion in *N. oceanica*, without impairing other physiological properties [116]. In addition, improved growth and lipid production were reported by overexpressing a basic helix–loop–helix TF NsbHLH2 in *N. salina*. Subsequently, nitrogen limitation at continuous cultivation led to an increased FA methyl ester production [117]. Moreover, it is revealed that NobZIP77 knockout fully preserves the cell growth rate and nearly triples TAG productivity in *N. oceanica* [118]. These tools enable gene-specific, mechanistic studies and have already allowed the engineering of improved *Nannochloropsis* strains with superior lipid production.

*Phaeodactylum tricornutum* is the diatom chassis for the production of a suite of natural and genetically engineered products [119]. In *P. tricornutum*, lipid production was elevated by the modification of either carbon partition or lipid synthesis. As for the carbon partition, (i) overexpression of a plastidial pyruvate transporter in *P. tricornutum* resulted in enhanced biomass, lipid contents, and growth [120]; and (ii) overexpression of *G6PDH* accompanied by high-CO_2_ cultivation resulted in a much higher amount of both lipid content and growth in *P. tricornutum* [121]. As for the lipid synthesis, (i) by knockout of *Δ9-DES*, EPA accumulation was increased by 1.4-fold in *P. tricornutum* [122]; and (ii) overexpression of *PtPAP* exhibited smaller plastoglobule as well as increased fucoxanthin compared to the *P. tricornutum* wild-type. The PUFAs (including EPA) were also increased [123]; and (iii) co-expression of *PtDGAT2B* and a *Δ5-FAE* resulted in higher lipid yields and enhanced levels of DHA in TAG [124]. Finally, elevated carbon partition and lipid synthesis were combined by co-expression of a malic enzyme a*Δ5*-*DES* in *P. tricornutum*. Neutral lipid content was remarkably increased by 2.4-fold, and EPA was significantly increased, too [125].

The species of *Chlorella* represents a highly specialized group of green microalgae that can produce high levels of lipids and protein. Many *Chlorella* strains can grow rapidly and achieve high cell density under controlled conditions and are thus considered to be promising lipid sources. Many advances in the genetic engineering of *Chlorella* have occurred in recent years, with significant developments in the successful expression of heterologous proteins for various applications [126]. A C-type bZIP TF HSbZIP1 was overexpressed in *Chlorella* sp. HS2, exhibiting increased FA production. [127]. Moreover, heterogeneous expression of *Arabidopsis thaliana* TF LEC1 significantly increased FA and lipid contents in *Chlorella ellipsoidea* [128]. In addition, in *Chlorella variabilis* NC64A, overexpression of *CvarLOG1* led to increased carbohydrate and lipid yield by approximately 30 and 20%, respectively [129].

In addition to the above-mentioned genus, many other microalgae have recently been engineered to produce enormous lipids. In *Ostreococcus tauri*, overexpression of ω3-desaturase altered the omega-3/omega-6 ratio in C16-PUFA and VLC-PUFA pools [130], while co-expression of two Δ6-desaturases prevented the regulation of C18-PUFA under phosphate deprivation and triggered glycerolipid fatty-acid remodeling, without causing any obvious alteration in growth or photosynthesis [131]. In *Neochloris oleoabundans*, *NeoLPAAT1*-overexpression exhibited a 1.9- and 2.4-fold increase in lipid and TAG contents [132]. Moreover, the co-expression of NeoLPAAT1 and NeoDGAT2 resulted in a 1.6- and 2.1-fold increase in total lipid and TAG content [133]. Furthermore, the co-expression of *LPAAT*, *GPAT*, and *DGAT* significantly enhanced the lipid accumulation in *N. oleoabundans* [134]. In addition, homogenous *LPAAT*-overexpression significantly increased TAG accumulation in *Cyanidioschyzon merolae*, too [135]. In *Schizochytrium* sp., the acetyl-CoA c-acetyltransferase was overexpressed to increase β-carotene and astaxanthin by 1.8- and 2.4-fold. On the other hand, three acyl-CoA oxidase genes were knocked out and the production of lipids was increased [136]. To elevate DHA contents in *Schizochytrium* sp., *CcME* and *MaELO3* were co-expressed; thus, DHA content was increased by 3.3-fold [137]. In *Dunaliella salina*, co-expression of *DsME1* and *DsME2* improved lipid production by up to 36.3% higher than the wild-type [138]. In *Scenedesmus* sp. and *Synechocystis* sp., compared to their wild types, overexpression of endogenous *ACCase* resulted in a 28.6% and 3.6-fold increase in lipid content, respectively [139,140]. 

**Table 3:** Genetic engineering of microalgae for enhanced lipid production.

Genus	Targeted Genes	Strategy *	Effect on Lipid Synthesis	References
*Chlamydomonas reinhardtii*	*GroELS*, *PEPC1*	OE, KD	Boosted lipids and lutein with 893 and 23.5 mg/L	[92]
	*SAMS*	OE	Two-fold increased lipid content	[93]
	*HpWS*	HE	150% and 39% increased astaxanthin and TAG content	[94]
	*FAX1*, *FAX2*, *ABCA2*	CE	2.4-fold increased TAG content	[95,96,97,98,99]
	*DOF*, *LACS2*, *CIS*	OE, KD	Lipids and FA content increased by 142% and 52%	[100,101]
	*MYB1*	OE	3.2-fold increased TAG content	[102]
	*FAT1*	OE	Increased lipid production	[103]
	*CpZF_CCCH1*	HE	Increased PUFA content by 16%	[104]
	*CrPrp19*	KD	1.3-fold increased TAG content	[105]
	*CrGPATer*	OE	Increased yield of OPO and galactolipids	[17]
	*ApACBP3*, *ApDGAT1*	HE	Increased C18:1 content by 59%	[107]
	*HpDGTT2*	HE	Enhanced TAG accumulation	[106]
*Nannochloropsis* spp.	*CrCAO*	HE	Increased lipid productivity	[109]
	*AtDXS*	HE	Lipids and TAG content increased by 111% and 149%	[110]
	*mCpTE*	HE	Elevated C12:0 content by 6.6-fold	[111]
	*FAD12*	OE	1.5-fold increase in EPA	[112]
	*No*Δ*6-FAE*	OE	Higher contents of FA, TAG and EPA	[50]
	*NoGPAT*, *AoGPAT*	OE	TAG, FA and PUFA increase by 51%, 42%, and 24%	[113]
	*NoPDAT*	OE	33% increased TAG content	[114,115]
	*NobZIP1*	OE	Elevation of lipid accumulation and lipid secretion	[116]
	*NsbHLH2*	OE	Increased FA production	[117]
	*NobZIP77*	KO	Double the peak productivity of TAG	[118]
	*NoDGAT2D*, *AtWRI1*, etc.	CE	Elevated MCT productivity by 64.8-fold	[16]
*Phaeodactylum tricornutum*	*PtDGAT2B, OtElo5*	CE	Higher lipid yields and TAG-associated DHA level	[124]
	*PAP*	OE	51% increased fucoxanthin content	[123]
	*G6PDH*	OE	Much higher of lipid and EPA content	[121]
	Δ*9-DES*	KO	1.4-fold increased EPA content	[122]
	*PtME*, *PtD5b*	OE	2.4-fold increased TAG content	[125]
	*PtPPT*	OE	30% increased lipid content	[120]
*Chlorella* spp.	*HSbZIP1*	OE	113% increased FA content	[127]
	*AtLEC1*	HE	Lipids and FA content increased by 30% and 33%	[128]
	*CvarLOG1*	OE	20% increased lipid yield	[129]
*Ostreococcus tauri*	*pω3-Des*	OE	Higher TAG-associated ALA	[130]
	Δ*6-DES*	OE	Increased TAG content	[131]
*Neochloris oleoabundans*	*NeoLPAAT1*, *NeoDGAT2*	CE	2.1- and 1.6-fold increased TAG and lipid content	[132,133]
	*LPAT, GPAT, DGAT*	CE	1.2-folds increase in FA content	[134]
*Cyanidioschyzon merolae*	*LPAT1*	OE	Increased TAG accumulation	[135]
*Schizochytrium* spp.	*AACT4419*	OE	1.8- and 2.4-fold increased β-carotene and astaxanthin	[136]
	*CcME, MaELO3*	CE	1.4-fold increased DHA content	[137]
*Dunaliella salina*	*DsME1*, *DsME2*	OE	36% higher lipid production	[138]
*Scenedesmus* sp. Z-4	*ACCase*	OE	29% increased lipid content	[139]
*Synechocystis* sp.	*ACCase*	HE	3.6-fold increased lipid content	[140]

* OE, overexpression; HE, heterogenous expression; CE, co-expression; KD, knockdown; KO, knockout.

## 6. Challenges and Perspectives

Currently, more health-promoting food and nutrients are required to satisfy global requirements [141]. Therefore, the integrated biorefinery has emerged as a reasonable approach for the production of high-value lipids [142]. In microalgae, the major challenge associated with high-value lipid production is low biomass, resulting in the high cost of cultivation and downstream processing [58]. Thus, it is necessary to develop approaches for an efficient system by improving the cultivation and energy-saving downstream processing of lipids. Economical lipid production can be realized by the upstream-downstream integration to reduce the processing cost. Consequently, energy and cost analysis should be performed to clarify the feasibility of the developed biorefinery in microalgae. The prospects should also comprise the metabolic engineering that is capable of high lipid and biomass production. Bioprocess strategies, together with metabolic engineering, will be promising for the development of engineering microalgae for food and nutraceutical applications.

It can be seen that the analysis of the lipid metabolism mechanism of eukaryotic algae by molecular biology technology has made great progress in the past ten years [143]. These advances have led to a growing interest in using algae for industrial purposes, such as nutrition or biofuels, driving much research [144]. Over the past decade, microalgae in Chlorophyta and Stramenopiles have been extensively studied and are considered commercially valuable algae [145]. We look forward to the future development of knowledge, which will undoubtedly happen. The fascinating and diverse biochemistry of algae can influence many fields around the globe.

## 7. Conclusions

Microalgae can produce a variety of bioactive compounds via biotechnology, yet the biological activity of many compounds has not been characterized. On the other hand, the mutant libraries have been greatly developed in microalgae, but for specific lipid compounds, the high-throughput detection, analysis, and separation technologies have not been followed up. In this way, the accurate analysis of active lipid compounds, as well as high-throughput screening via mass spectrometry, fluorescence, and microfluidic technologies need to be developed in the future.

Currently, microalgae are regarded as a potential platform for green lipid production. Here, we reviewed the progress of genetic engineering to improve lipid production of microalgae in the past five years. Most of the engineering strategies involved the modification of a single metabolic pathway by introducing carbon into lipid synthesis or enhancing carbon capture. Despite several efforts to improve lipid accumulation in transgenic microalgae, for now, the ability of microalgae to produce high-value lipids is not enough. To further increase lipid production, engineering strategies must simultaneously improve photosynthetic responses and channel carbon flux to lipids, without limiting the growth of the host species. Future research is suggested to focus on microalgal species that can produce high-value lipids in large-scale productivity. The robust lipid species plus rational approaches of engineering are expected to lead us to an amazing world of microalgae, with highly elevated lipid productivity and profiles.
